# Wheat *TaPUB1* modulates plant drought stress resistance by improving antioxidant capability

**DOI:** 10.1038/s41598-017-08181-w

**Published:** 2017-08-08

**Authors:** Guangqiang Zhang, Meng Zhang, Zhongxian Zhao, Yuanqing Ren, Qinxue Li, Wei Wang

**Affiliations:** 1 State Key Laboratory of Crop Biology, Shandong Key Laboratory of Crop Biology, College of Life Sciences, Shandong Agricultural University, Tai’an, Shandong 271018 P.R. China; 2 Collaborative Innovation Center, Jining Medical University, Jining, Shandong 272067 P.R. China

## Abstract

E3 ligases play significant roles in plant stress tolerance by targeting specific substrate proteins for post-translational modification. In a previous study, we cloned *TaPUB1* from *Triticum aestivum* L., which encodes a U-box E3 ligase. Real-time polymerase chain reaction revealed that the gene was up-regulated under drought stress. To investigate the function of *TaPUB1* in the response of plants to drought, we generated transgenic *Nicotiana benthamiana* (*N*. *benthamiana*) plants constitutively expressing *TaPUB1* under the *CaMV35S* promoter. Compared to wild type (WT), the transgenic plants had higher germination and seedling survival rates as well as higher photosynthetic rate and water retention, suggesting that the overexpression of *TaPUB1* enhanced the drought tolerance of the *TaPUB1* overexpressing (OE) plants. Moreover, less accumulation of reactive oxygen species (ROS) and stronger antioxidant capacity were detected in the OE plants than in the WT plants. To characterize the mechanisms involved, methyl viologen (MV) was used to induce oxidative stress conditions and we identified the functions of this gene in the plant tolerance to oxidative stress. Our results suggest that *TaPUB1* positively modulates plant drought stress resistance potential by improving their antioxidant capacity.

## Introduction

In the natural environment, plants are consistently exposed to a series of unfavorable conditions that affects their growth and development. Over the course of evolution, plants have acquired complex and comprehensive mechanisms to withstand these unfavorable stress conditions^1^; these include sophisticated physiological and biochemical regulatory networks^2^. The major mechanisms involved in adaptation to water stress include reduced water loss, enhanced water uptake and its efficient use, and production of low-molecular-weight osmolytes that sustain cellular functions under drought conditions. A number of antioxidants and enzymes scavenge the reactive oxygen species, such as superoxide and singlet oxygen, which are generated due to water deficit. At the molecular level, several drought-inducible genes and transcription factors function in establishing the stress tolerance network in plants^3–5^.

The ubiquitin-26S proteasome system (UPS) is an extremely complex and versatile system for protein degradation that plays important roles in the response of all eukaryotic species to varied environmental conditions, such as those of drought and salt stress^6, 7^. In recent years, data have shown that the UPS targets many intracellular regulators that play key roles in hormone signaling pathways, responses to abiotic stresses, and in battling pathogens^8^. The ubiquitination process involves the concerted action of an intricately linked network of enzymes, namely E1, E2, and E3. Of these three proteins, the E3 ubiquitin ligases are the most diverse proteins in the UPS that specifically recognize an extensive range of targets^9^. Depending on the type of substrate, E3 ubiquitin ligases can be subdivided into the following kinds: F-box type, RING type, U-box type, and HECT type^10^.

The U-box type of E3 ubiquitin ligase has a highly conserved domain of 70 amino acids that forms a scaffold structure. The plant U-box (PUB) proteins contain some distinct categories, implying that they have a wide array of roles. Lack or mutation of the U-box has been demonstrated to abolish the ubiquitination activity^11, 12^. In recent years, it has been reported that some U-box proteins participate in the organism’s response to hormones^13, 14^, and tolerance to biotic^15, 16^ and abiotic stresses^17, 18^.

Wheat is the third largest crop in the world and is consumed by more than 35% of the world population. The main constraints in wheat cultivation are the scarcity of planting area and water. Therefore, studies on drought tolerance and resistance are very important for wheat production^19, 20^. In a previous study, we cloned a U-box protein gene from wheat and named it *TaPUB1*. Overexpression of *TaPUB1* could confer enhanced salt tolerance in transgenic *N*. *benthamiana*. Moreover, drought stress could induce the expression of *TaPUB1*. In this study, we present and discuss the results of our investigation on the physiological and molecular mechanisms of increased drought tolerance upon *TaPUB1* overexpression.

## Results

### Selection of *35S::TaPUB1* transgenic *N*. *benthamiana* plants and detection of E3 ligase activity

**Figure 1:**
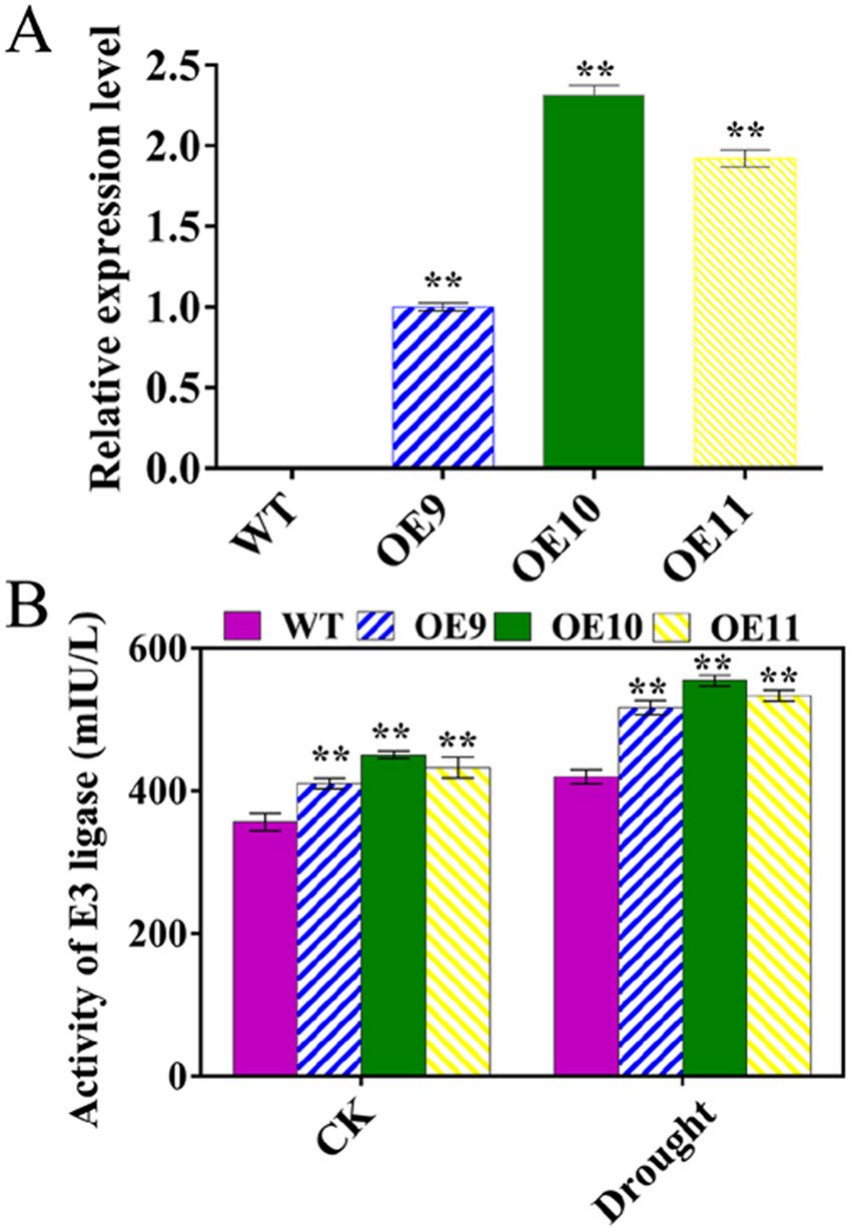
Confirmation of *TaPUB1* expression in transgenic *N*. *benthamiana* lines. (**A**) The relative expression levels of *TaPUB1* in the WT plants and three different *TaPUB1* transgenic lines (OE9, OE10, and OE11). (**B**) The E3 ligase activity in leaves of OE and WT plants. The experiments were repeated three times and the bars indicate SEs. * and ** indicate significant differences at P < 0.05 and P < 0.01 in the values among the three overexpression lines and the WT plants.

In our previous study, the full-length cDNA of *TaPUB1* (GenBank ID: JX307854) was obtained from *Triticum aestivum* L. ‘HF9703’. In this research, we chose plants from three *35S::TaPUB1* transgenic *N*. *benthamiana* lines (OE9, OE10, OE11) and WT to study the resistance of the OE plants to drought. The *TaPUB1* transgenic plants were authenticated by qRT-PCR. All the three independent homozygous lines showed different levels of expression of *TaPUB1* whereas no expression was observed in WT (Fig. 1A). We also measured the E3 ligase activity and its response to drought stress in the leaves of OE and WT lines. Drought stress was observed to increase E3 ligase activity in all the plants; the activity was higher in the OE plants than in the WT under both normal and drought conditions (Fig. 1B). These results provided evidence that *TaPUB1* had stable expression in the *N*. *benthamiana* plants and increased the activity of E3 ligase substantially.

### Reduced sensitivity of germination of *TaPUB1* overexpressing *N*. *benthamiana* seeds to drought stress

**Figure 2:**
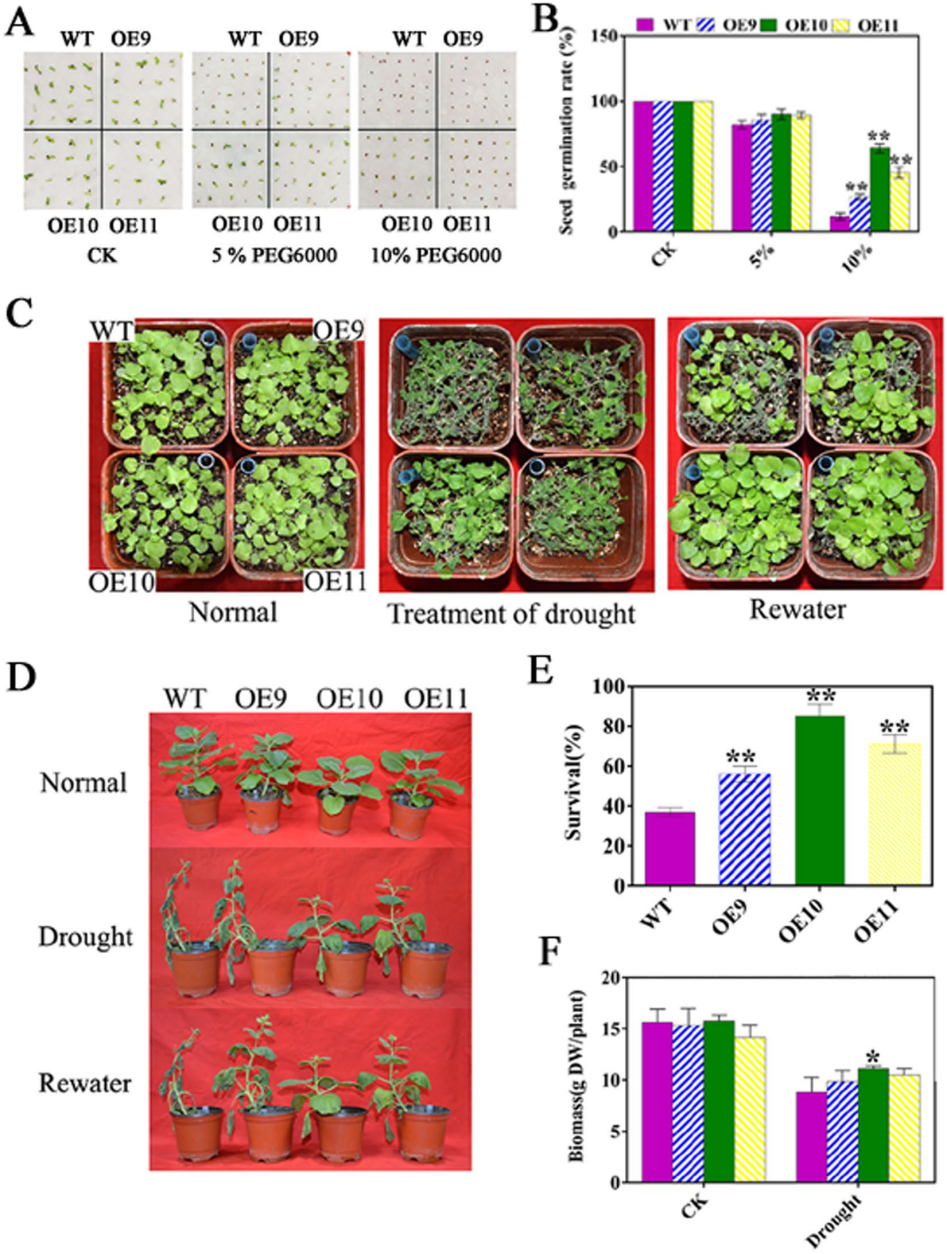
Influence of drought stress on the growth of *TaPUB1*-overexpressing and WT plants. (**A**) Phenotype of seedlings grown on MS medium containing different concentrations of PEG6000 for 7 d. (**B**) Comparison of the germination rates of *TaPUB1*-overexpressing and WT plants. Values are means ± SEs of at least three independent experiments (n = 45 seeds per treatment). (**C**) The phenotypes of 2-week-old *TaPUB1*-overexpressing and WT plants subjected to drought treatment for 5 d and to subsequent re-watering for 2 d. (**D**) The survival ratio was calculated as the percentage of plants that resumed growth 2 d after re-watering. (**E**) Phenotypes of OE and WT plants exposed to water withholding treatment at the 2-month-old stage. Representative plants subjected to drought stress for 5 d. After 5 d of drought treatment, the plants were rewatered and their recovery was recorded. (**F**) The biomass of *TaPUB1*-overexpressing and WT plants. The experiments were repeated three times and the bars indicate SEs. * and ** indicate significant differences at P < 0.05 and P < 0.01 in the values among the three overexpression lines and the WT plants.

The expression of *TaPUB1* was upregulated following PEG6000 treatment. Thereafter, the function of *TaPUB1* in response to drought stress was investigated using the three *TaPUB1* **-**overexpressing lines. Firstly, the percentage of seed germination in the transgenic and WT plants under water stress conditions was determined. The seeds of WT plant and the three *TaPUB1*-overexpressing lines were germinated on petri dishes containing water or PEG6000 (Fig. 2A). In the absence of PEG6000, the germination of seeds from the OE lines was comparable with those from the WT plants. In the presence of 5% PEG6000, we determined slight difference in germination rates between WT and OE seeds. However, the germination percentage in the case of transgenic lines was distinctly higher than that in the WT plants on the medium containing 10% PEG6000 (Fig. 2A,B). These results provide evidence that during the seed germination stage, the transgenic plants were less sensitive to drought stress than were the WT plants.

### Overexpression of *TaPUB1* enhanced the drought tolerance of transgenic seedlings and adult *N*. *benthamiana* plants

The OE and WT seeds were sown in roseite and after two weeks of growth of the seedlings, watering was withheld for 5 d. After this drought stress, the seedlings were rewatered for 2 d. The survival rates of transgenic plants exposed to this treatment ranged between 56% and 81% whereas that of WT was 37% (Fig. 2C,E).

**Figure 3:**
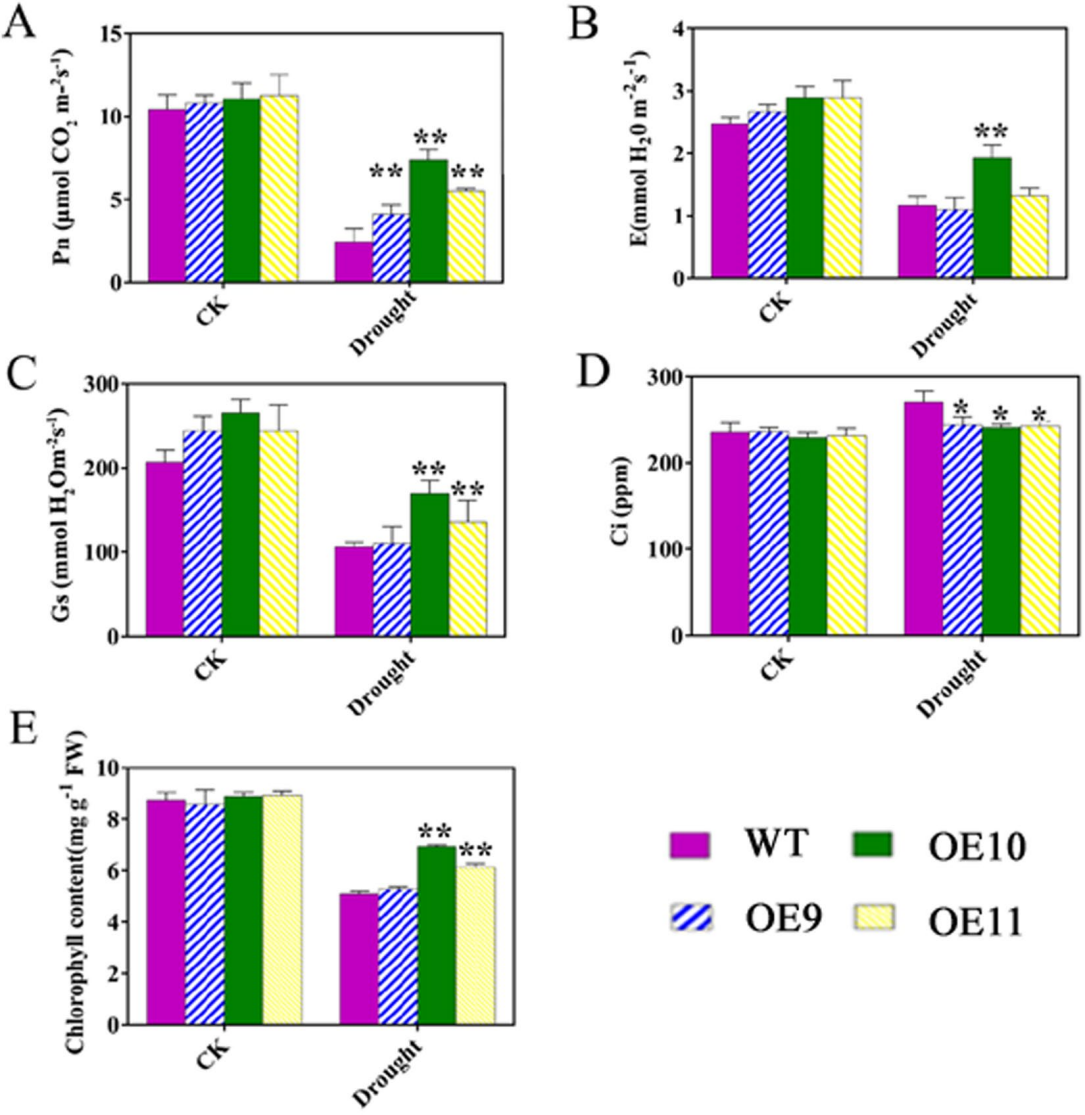
Photosynthetic performance of *TaPUB1*-overexpressing lines and WT plants after drought treatment. (**A**) Net photosynthetic rate, Pn. (**B**) Transpiration rate, E. (**C**) Stomatal conductance, Gs. (**D**) Intercellular CO_2_ concentration, Ci. **(E)** Chlorophyll content. The experiments were repeated three times and the bars indicate SEs. * and ** indicate significant differences at P < 0.05 and P < 0.01 among the three overexpression lines and the WT plants.

**Figure 4:**
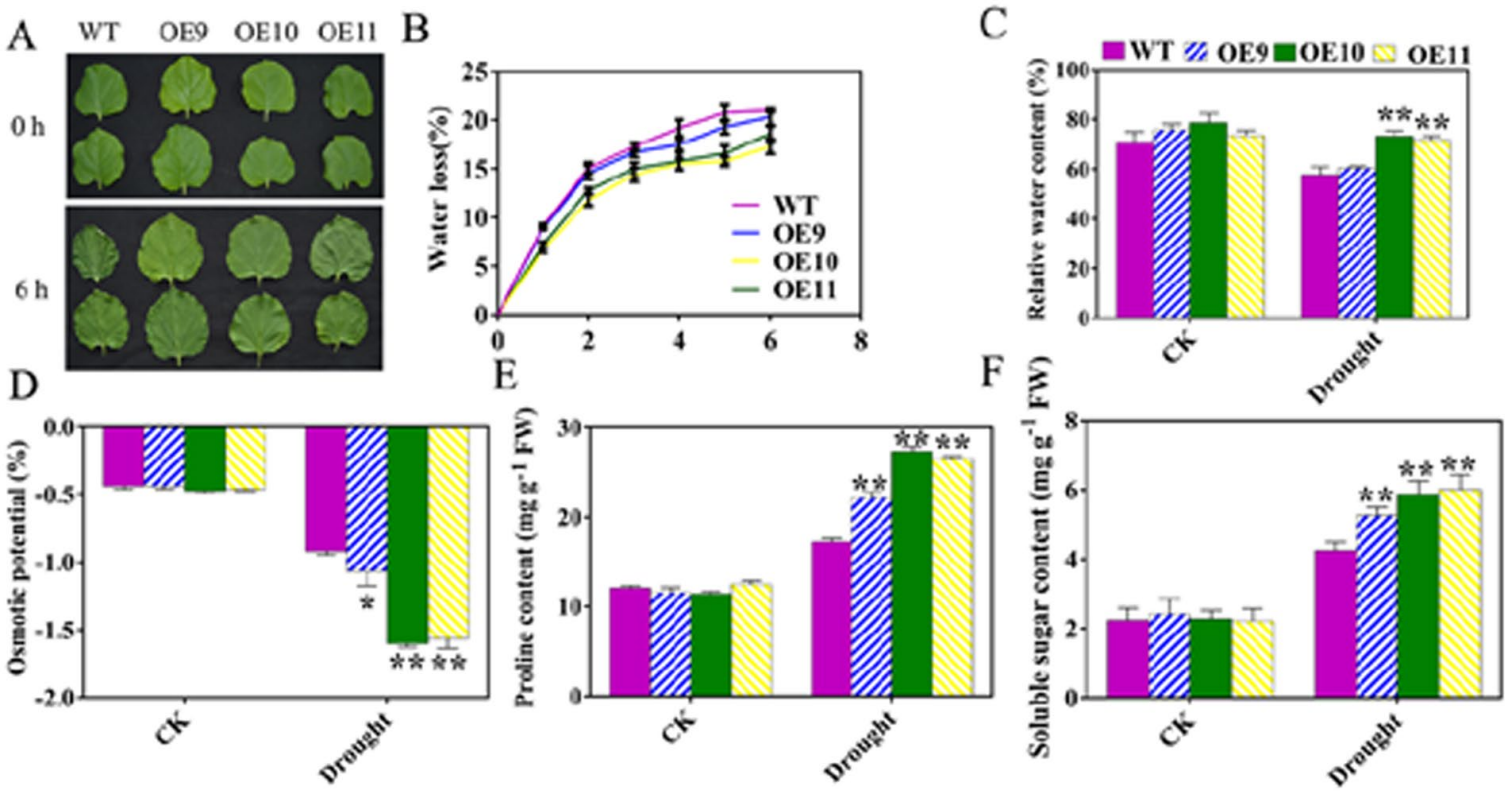
Influence of drought stress on water loss, relative water content, osmotic potential, proline content, and soluble sugar content in the OE and WT plants. The phenotypes and kinetics of water loss from the leaves of 2-month-old *N*. *benthamiana* lines (**A**,**B**). (**C**) Relative water content. Blades separated from 2-month-old *TaPUB1*-overexpressing and WT lines after drought treatment. (**D**) Osmotic potential. (**E**) Proline content and (**F**) Soluble sugar content after drought treatment. The experiments were repeated three times and the bars indicate SEs. * and ** indicate significant differences at P < 0.05 and P < 0.01 among the three overexpression lines and the WT plants.

Furthermore, two-month-old *N*. *benthamiana* plants were allowed to dehydrate naturally for 5 d, and were subsequently rewatered for 3 d. After this drought treatment, the *TaPUB1*-overexpressing plants revealed more robust phenotypes, including higher biomass, than the WT plants (Fig. 2D,F). To further validate the drought tolerance of OE plants, two-month-old transgenic lines and WT plants were subjected to drought stress for 5 d, and the photosynthetic parameters of the plants were examined using CIRAS-2 (Hitchin, UK) photosynthesis system. The results showed that drought stress resulted in a significant decline in the net photosynthetic rate (Pn), transpiration rate (E), and stomatal conductance (Gs) in both the OE and WT plants (Fig. 3A–C). However, the Pn in the OE plants was higher than that in the WT plants (Fig. 3A) under drought treatment. Similar results were observed for E (Fig. 3B) and Gs (Fig. 3C). The chlorophyll content of the OE lines was also higher than that of the WT plants under stress conditions (Fig. 3E). However, the intercellular CO_2_ concentration (Ci) was lower in the transgenic plants than it was in the WT plants (Fig. 3D). Overall, the results depicted in Figs 2, 3 and 4 indicate that overexpression of *TaPUB1* increased the resistance to drought in plants.

### Overexpression of *TaPUB1* increased the water retention capacity of OE lines

To determine the water retention capacity of transgenic *N*. *benthamiana* plants, we measured the rate of water loss, relative water content (RWC), osmotic potential, and the accumulation of compatible solutes, including proline and soluble sugars. The detached leaves of OE plants were observed to have lost less water than those of WT plants within 6 h of dehydration (Fig. 4A,B). There were no significant differences in the leaf RWC among the WT and OE lines under water-replete condition. In contrast, after drought the RWC in OE plants was higher than that in the WT plants (Fig. 4C). Moreover, the osmotic potential of the transgenic plants was lower than that of the WT plants (Fig. 4D) after the drought treatment. At the same time, the contents of proline and soluble sugars were higher in the OE plants than in the WT plants (Fig. 4E,F). This suggests that the water retention ability of the transgenic lines was better than that of the WT plants.

### Influence of drought on electrolyte leakage, MDA content, and protein carbonylation in the *TaPUB1*-overexpressing plants

**Figure 5:**
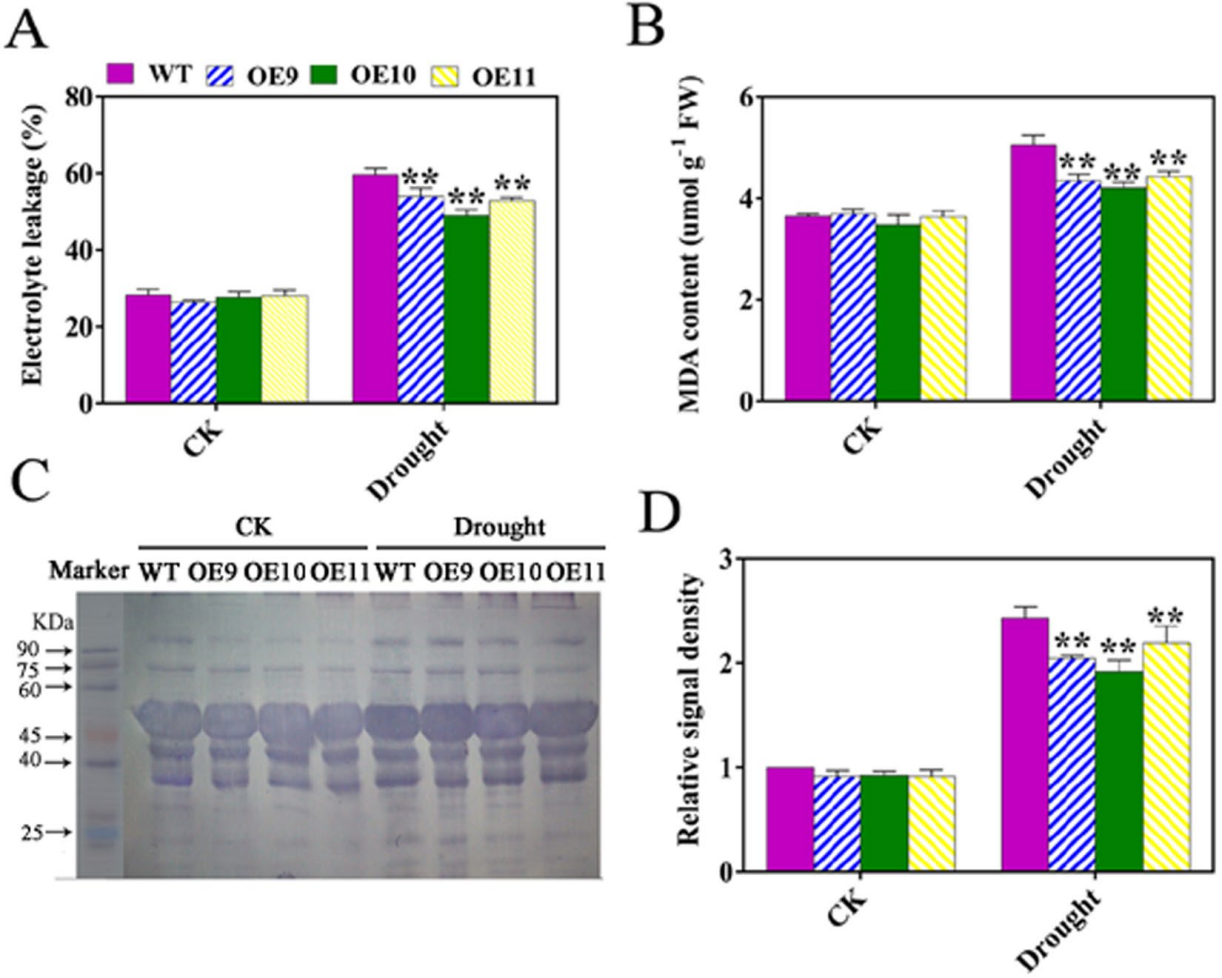
Influence of drought stress on electrolyte leakage, malondialdehyde (MDA), and protein carbonylation levels in the OE and WT plants. (**A**) Electrolyte leakage. (**B**) MDA content. (**C**) Protein carbonylation levels. (**D**) Relative signal density of protein carbonylation levels observed in (**C**). The experiments were repeated three times and the bars indicate SEs. * and ** indicate significant differences at P < 0.05 and P < 0.01 among the three overexpression lines and the WT plants.

When a plant tissue is subject to stresses, the membrane structure of its cells is impaired, which leads to the increase in the membrane permeability and the electrolyte leakage is also increased, consequently. The electrolyte leakage levels in the OE and WT plants were similar in the absence of drought stress. However, the transgenic plants maintained lower levels of electrolyte leakage that those in the WT plants after the drought treatment (Fig. 5A). The MDA content of the OE and WT plants increased after the drought treatment, but the increase was less in the OE plants than that in the WT plants (Fig. 5B). These results indicate that the membrane injury in the transgenic plants was less than that in the WT plants.

All the amino acids are sensitive to oxidative stress. The protein carbonyl content increases significantly after oxidative modification. The protein carbonyl content can be used as an indicator of protein oxidative damage^21^. The protein carbonyl levels of the transgenic and WT plants were detected by immunoblotting. Although the protein carbonyl content increased in both the transgenic and the WT plants after drought treatment (Fig. 5C,D), the levels in the transgenic plants were observably lower than that in the WT plants. Overall, these results showed that the damage in the OE plants was less than that in the control plants after the drought treatment (Fig. 5).

### Overexpression of *TaPUB1* reduced the accumulation of ROS in the transgenic *N*. *benthamiana* plants under drought stress

**Figure 6:**
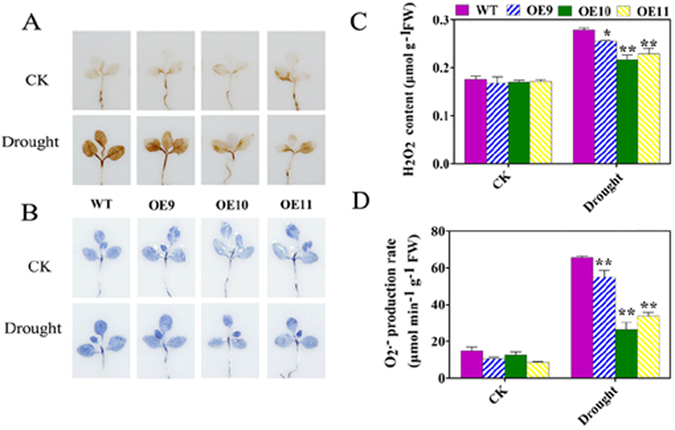
Influence of drought stress on ROS accumulation in the OE and WT plants. *In situ* detection of H_2_O_2_ (**A**) and ${{\rm{O}}}_{2}^{\cdot -}$ (**B**) by DAB and NBT staining, respectively, in *TaPUB1* overexpression and WT seedlings grown on roseite medium for two weeks and treated with 100 mM mannitol, thereafter, for 7 d. **(C)** H_2_O_2_ content and **(D)** ${{\rm{O}}}_{2}^{\cdot -}$ production rate. The experiments were repeated three times and the bars indicate SEs. * and ** indicate significant differences at P < 0.05 and P < 0.01 among the three overexpression lines and the WT plants.

The production and accumulation of ROS can be induced by drought treatment. We examined the levels of endogenous hydrogen peroxide (H_2_O_2_) by 3,3′-diaminobenzidine (DAB) staining and those of superoxide radical (${{\rm{O}}}_{2}^{\cdot -}$) by nitrotetrazolium blue chloride (NBT) staining. After imposition of drought stress, different levels of H_2_O_2_ were detected in the seedlings of the WT and transgenic lines; however, the formation of brown precipitate in WT plants was much more than in the transgenic lines (Fig. 6A). Similar results were observed for ${{\rm{O}}}_{2}^{\cdot -}$ production (Fig. 6B). The quantitative results of H_2_O_2_ content and ${{\rm{O}}}_{2}^{\cdot -}$ production rate were consistent with the results of staining (Fig. 6C,D). These results suggested that the overexpression of *TaPUB1* reduced the accumulation of ROS in the OE lines.

Antioxidant enzymes can effectively remove ROS. The UPS is also involved in clearance of oxidized proteins from cells^22^. We evaluated the activity of four antioxidant enzymes, including superoxide dismutase (SOD), peroxidase (POD), catalase (CAT), and ascorbate peroxidase (APX) in the transgenic and WT plants (Fig. S1). There were no obvious differences in the activities of the four antioxidant enzymes mentioned above, among the plants under normal conditions. However, under drought stress, the activities of all the antioxidant enzymes in the transgenic plants were significantly higher than those in the WT plants.

### *TaPUB1* overexpression alters the expression of oxidation- and drought-related genes

**Figure 7:**
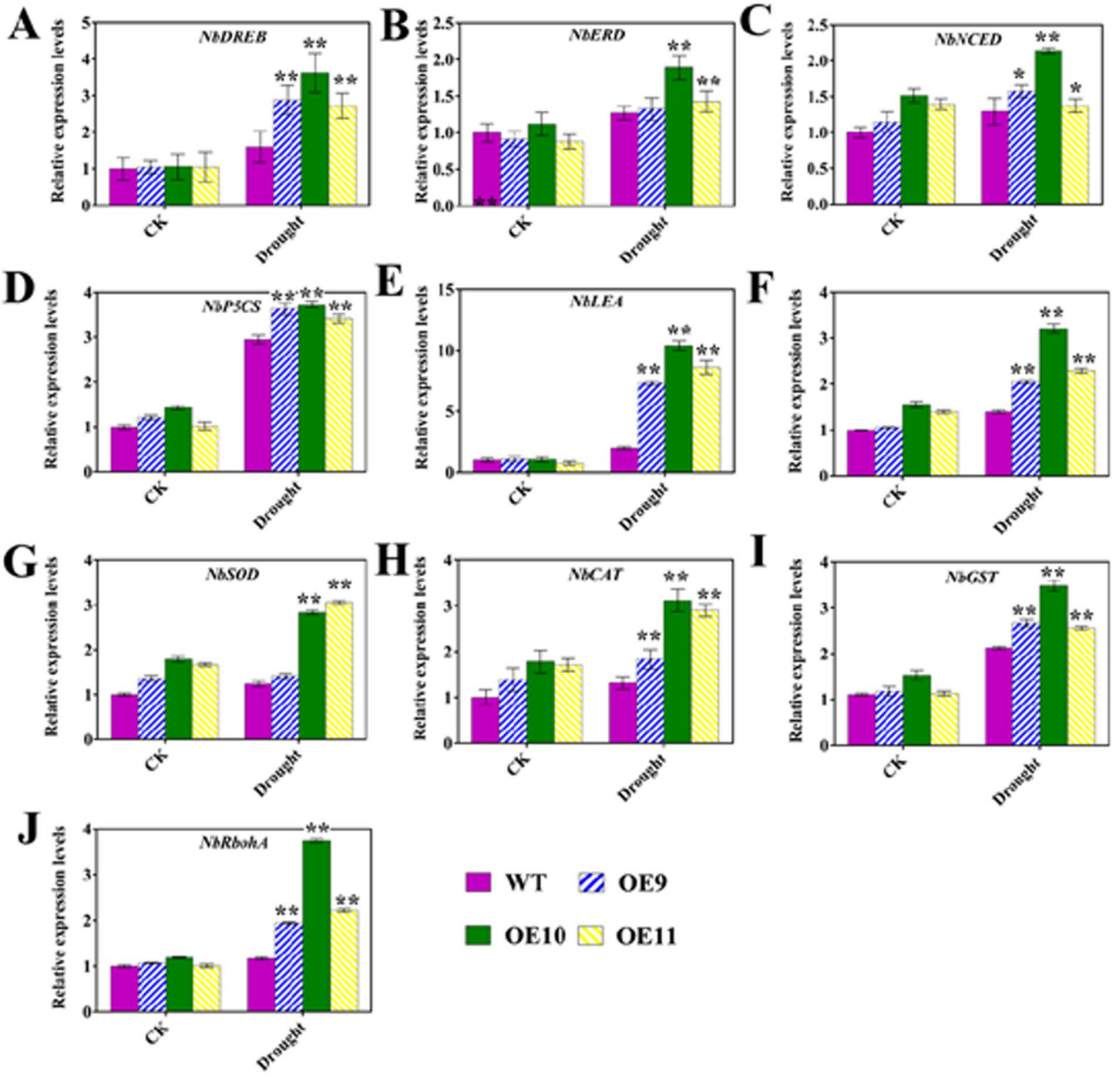
Expression of some antioxidant-and drought-related genes in OE and WT plants under drought stress. The histograms indicate the expression levels of the mentioned genes **(A**–**J)** in the transgenic and WT plants relative to their levels in the WT plants, which were considered as 1. Expression was normalized to the level of expression of actin in each sample was taken as the control. The experiments were repeated three times and the bars indicate SEs. * and ** indicate significant differences at P < 0.05 and P < 0.01 in the values among the three overexpression lines and the WT plants.

To further elucidate the possible molecular mechanisms of the increased drought tolerance in the transgenic *N*. *benthamiana* plants, we performed qPCR analysis of a set of known antioxidant- and drought-related genes in *N*. *benthamiana* plants; the genes whose expression were assessed included dehydration-responsive element binding (*NbDREB*), early responsive to dehydration (*NbERD*), 9-cis-epoxycarotenoid dioxygenase (*NbNCED*), late embryogenesis-abundant protein (*NbLEA*), pyrroline-5-carboxylate synthase (*NbP5CS*), and antioxidant-related genes. including ascorbate peroxidase *(NbAPX)*, superoxide dismutase (*NbSOD*), catalase (*NbCAT*), glutathione S-transferases (*NbGST*), and respiratory burst oxidase homolog A (*NbRbohA*). The expression of drought-related genes in the transgenic and WT plants showed varying degrees of upregulation after drought treatment (Fig. 7A–J). However, the increase in expression was higher in the OE plants than that in the WT plants. Therefore, the expression levels of all these stress-inducible marker genes were up-regulated by drought stress and by overexpression of *TaPUB1* in *N*. *benthamiana* plants.

### *TaPUB1* overexpression improved the antioxidant capacity of transgenic *N*. *benthamiana* lines

The results depicted in Figs 6 and S1 showed that the overexpression of *TaPUB1* alleviated the accumulation of ROS and enhanced the antioxidant capacity of the transgenic plants under drought stress. Oxidative stress is a common secondary stress in almost all kinds of stress conditions^23^ and is also the primary mechanism by which biotic and abiotic stresses influence and regulate the biological metabolism^24^. To study the involvement of the antioxidant ability in the improved drought tolerance of the *TaPUB1*-overexpressing *N*. *benthamiana* plants, we simulated oxidative stress in plants by applying methyl viologen (MV).

**Figure 8:**
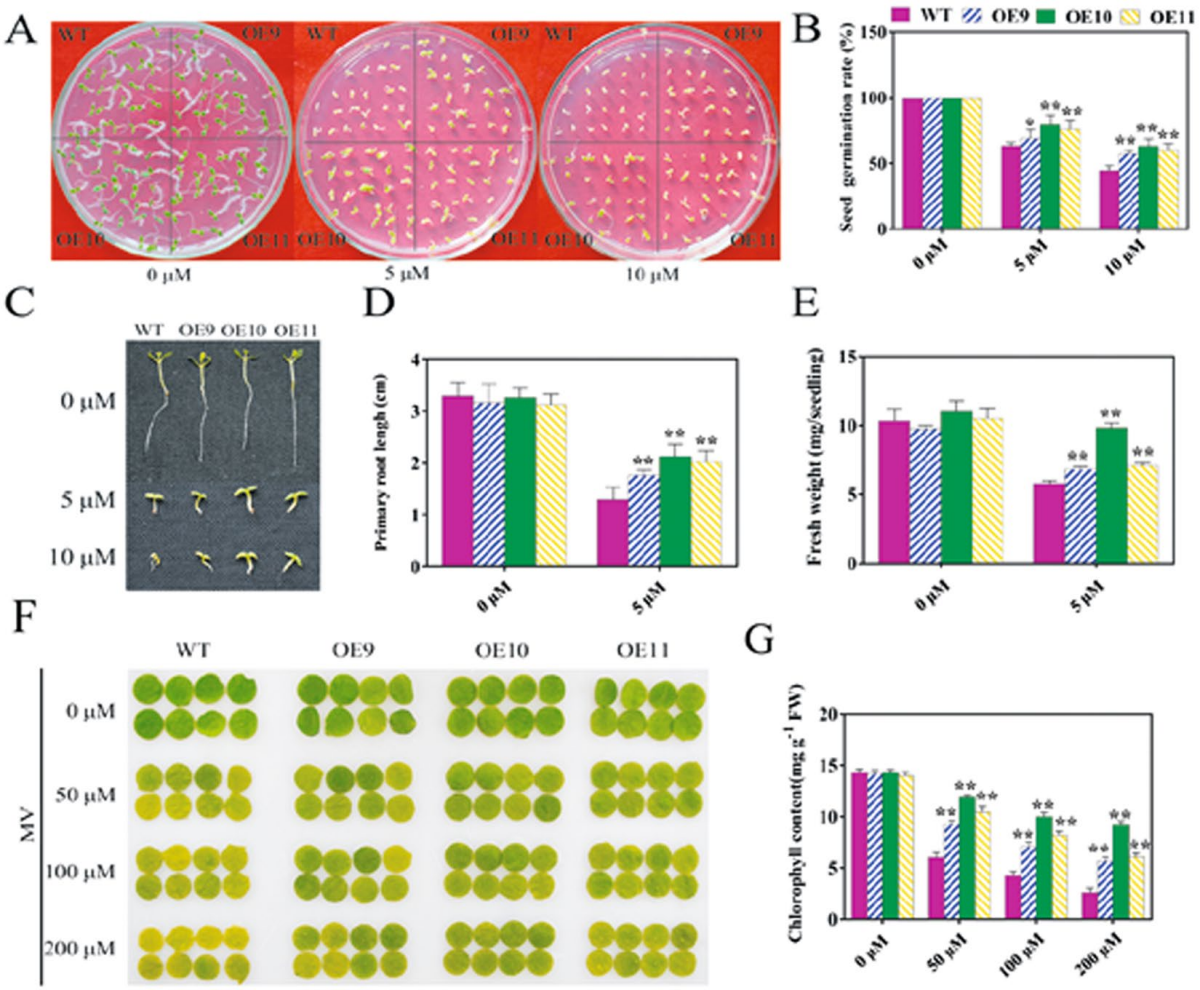
Analysis of oxidative stress tolerance in *TaPUB1-*overexpressing *N*. *benthamiana* plants. (**A**) Germination of seeds on MV-free or MV-containing MS culture medium for 2 weeks. (**B**) Germination rates of the transgenic and WT seedlings under normal conditions and oxidative treatment for 7 d. (**C**) Phenotype of seedlings of each line after 14 d. (**D**) Root length and (**E**) Fresh weight were obtained from seedlings that were transferred to MV-containing MS medium. (**F**) Phenotype of leaf discs after MV treatment. (**G**) Chlorophyll content in the leaf discs after MV treatments. The experiments were repeated three times and the bars indicate SEs. * and ** indicate significant differences at P < 0.05 and P < 0.01 in the values among the three overexpression lines and the WT plants.

The transgenic and WT seeds were sown in MV-free or MV-containing Murashige and Skoog’s (MS) culture medium. In the MV-free MS medium, the seeds of the *TaPUB1*-overexpressing lines showed similar germination rates to those of the WT plants. However, the number of seedlings with green cotyledons was higher in the transgenic plants than that in the WT on medium containing MV (Fig. 8A). The statistical analysis showed that the transgenic seeds had higher germination rate than the WT seeds (Fig. 8B).

We next assayed the growth responses of the OE seedlings to oxidative stress. Under normal conditions, the root morphology and length were similar in both the WT and transgenic lines, whereas in the presence of MV, the root was longer in the *TaPUB1* overexpressing plants than that in the WT plants (Fig. 8C). The root of the *TaPUB1* overexpressing lines was 18% and 34% longer than that of the WT plants in the presence of 5 and 10 µM MV, respectively (Fig. 8D). Similar results were obtained for the fresh weight of seedlings (Fig. 8E).

We placed leaf discs from 2-month-old plants in Petri dishes containing different concentrations of MV. The exposure to different concentrations of MV for 3 d resulted in the manifestation of chlorosis in the leaf discs of the WT and OE plants; however, the leaf discs of the WT displayed much more chlorotic spots than those of the OE plants (Fig. 8F). This was consistent with the results of the chlorophyll content (Fig. 8G).

We further detected the accumulation of ROS in the leaves of the transgenic and WT plants after 12 and 24 h of MV treatment. The results showed that a considerably lower amount of ROS accumulated in the *TaPUB1*-overexpressing plants than in the WT plants (Fig. S2A,B). The quantitative results of the H_2_O_2_ content and ${{\rm{O}}}_{2}^{\cdot -}$ production rates were similar to the results of staining (Fig. S2C,D). In addition, the activities of CAT and POD in the transgenic plants were significantly higher than those in the WT plants under oxidative stress (Fig. S2E,F).

The results described in Figs 8 and S2 suggest that *TaPUB1* overexpression enhanced the oxidative stress tolerance of the OE plants.

## Discussion

The PUB(U-box) proteins, as members of the E3 ligase family, have many important biological functions^17, 25, 26^. In a previous study, we obtained a U-box protein gene, *TaPUB1*, from wheat by homologous cloning. In order to find its functions in abiotic stress tolerance, we constructed the overexpression vector of *TaPUB1* and generated heterologous overexpression *N*. *benthamiana*. We selected three independent homozygous transgenic T3 lines (OE9, OE10, OE11) with different *TaPUB1* expression levels (Fig. 1A) for this study. Many U-box-containing proteins have been shown to have E3 ligase activity^25^. Sequence alignment and phylogenetic tree analysis indicated that *TaPUB1* might encode a U-box protein. In the present study, the higher levels of E3 ligase activity were observed in the OE lines than that in the WT plants under both normal and drought conditions (Fig. 1B), suggesting the role of TaPUB1 in E3 activity.

The structures and functions of the PUB E3 family members have been identified and analyzed in several plants^27, 28^. Researches had shown that U-box proteins play important roles in the response of plants to abiotic stresses^29^. For instance, *AtCHIP* is involved in response to temperature stress in *Arabidopsis* ^30^. *OsPUB15* has an important effect in alleviating cellular oxidative stress^31^. Soybean E3 ubiquitin ligase, *GmPUB8*, is involved in negative regulation of drought response in *Arabidopsis* ^18^. In this research, the transcript levels of *TaPUB1* were also up-regulated by drought stress, suggesting that *TaPUB1* may be involved in the plant drought stress response.

The effects of water stress range from morphological to molecular levels and are obvious at all phenological stages of plant growth whenever the drought takes place^5^. Seed germination is the first critical step in plant growth and development and water stress significantly delays the onset and reduces the rate of seed germination^32, 33^. From Fig. 2, it is clear that the transgenic lines had significantly higher germination rates than the WT lines under PEG-induced water stress. Water shortage has a very large impact both on seedling and vegetative stages. The improved survival rates, more robust phenotypes, including more green leaves and higher biomass of transgenic plants were observed under drought stress (Fig. 2). These results indicate that *TaPUB1* plays a positive role in plant drought tolerance.

Photosynthesis is one of the key processes affected by water deficit because of the decreased CO_2_ diffusion to the chloroplast and metabolic constraints^34, 35^. Our data showed that the OE lines maintained higher Pn and chlorophyll contents than did the WT plants after the drought treatment (Fig. 3A,C,E). Although the Gs (Fig. 3C) of transgenic leaves was higher than WT under drought stress, which resulting in more CO2 accumulation inside the stroma of the chloroplast, however, the Ci (Fig. 3D)of the transgenic plants was lower than WT since their higher Pn (Fig. 3A). These results also suggest the improved drought stress tolerance by overexpression of *TaPUB1*.

Drought stress substantially decreased the water contents of the plant tissue, which had pronounced effects on photosynthetic rate and plant growth^36^. Our results showed that, compared to WT, the OE lines had lower water loss rate and higher RWC (Fig. 4A–C), this may be related to the lower osmotic potential (Fig. 4D) although the transpiration rate of the transgenic plants was higher (Fig. 3B) than the WT plants. Plants can accumulate compatible osmolytes to adjust the intracellular osmotic potentials in response to drought stress. Proline and soluble sugars, as compatible osmolytes, are thought to function mainly in osmotic adjustment and crucial to sustain cellular functions under drought stress^5, 37^. The high water retention ability in the OE lines might partially be a result of the increase in the contents of compatible osmolytes (Fig. 4E and F).

Drought stress can inhibit the photosynthetic activity in tissues due to an imbalance between light capture and its utilization^38^. Down regulation of photosystem (PS) II activity results in changes in the quantum yield. These changes in the photochemistry of chloroplasts in the leaves of drought-stressed plants result in the dissipation of excess light energy in the PSII core and antenna, thus generating ROS, which are potentially dangerous under drought stress conditions^39^. ROS plays a crucial role in causing cellular damage under drought stress^40^. Injury caused by ROS to biological macromolecules under drought stress is among the major deterrents to plant growth^5^. The membranes of plant cells are the main targets of oxidative damage induced by drought stress, and cell membrane stability has been widely used for the estimation of stress tolerance^41^. The degree of injury to cell membrane induced by dehydration stress can be evaluated by detection of the electrolyte leakage from the leaves, and the intracellular MDA content is commonly used as an indicator of the degree of lipid peroxidation^42, 43^. The transgenic plants maintained lower levels of electrolyte leakage and MDA content than did the WT plants after the drought treatment (Fig. 5A and B). ROS can attack most of the biological macromolecules, such as intracellular proteins, and damage their function^40^. There are less accumulation of protein carbonyl content in the transgenic plants (Fig. 5C and D) than that in WT. These results demonstrated that the *TaPUB1* overexpression alleviated the oxidative damage in plants.

Furthermore, low levels of H_2_O_2_ and ${{\rm{O}}}_{2}^{\cdot -}$ were detected in the OE plants under drought stress (Fig. 6). The low level of ROS in the OE plants was due to the higher activity of antioxidant enzymes, such as SOD, POD, CAT, and APX (Supplementary Fig. S1). The higher levels of proline and soluble sugars in the OE plants might also have participated in ROS scavenging^37^. These results suggest that the enhanced drought stress tolerance of OE plants maybe associate with the active oxygen scavenging system.

Drought can trigger a series of plant responses, including change in the level of gene expression^44^. These genes play a role in the stress response and function in stress tolerance at the molecular level^4^. Hence, we detected the expression of some of the antioxidant- and drought-related genes. As shown in Fig. 7, the expression levels of all these stress-inducible marker genes were increased by drought stress, and a larger increase was observed in the OE plants than in the WT plants. This suggest that the improved stress tolerance of OE plants might be related to the changed expression of stress-related genes, but the underlying mechanism is unclear.

Many stresses, such as salt and drought stress, disrupt the cellular homeostasis by enhancing the production of ROS^45^. Therefore, oxidative stress is a secondary stress involved in almost all the environment stresses^46^. The overexpression of *TaPUB1* decreased the oxidative damage and enhanced the activity of antioxidant enzymes in the transgenic plants under drought stress conditions (Figs 5 and 6 and S1). Further, we detected the antioxidative compete of the transgenic lines in the present study, MV was used to induce oxidative stress. The OE plants had higher seed germination rates and longer roots than the WT plants under oxidative stress condition (Fig. 8A–E). At the same time, the leaf disc experiment also suggested a lower damage in the transgenic plants than that in the WT plants under oxidative stress (Fig. 8F and G). The results shown in Supplementary Fig. S2 indicated the less ROS accumulation and higher antioxidant activity in the OE plants than that in the WT plants after MV treatment. Therefore, the overexpression of *TaPUB1* improved the oxidative stress tolerance of the OE lines, which might be involved in their drought stress tolerance.

In conclusion, the data obtained in this study clearly suggest that overexpression of the U-box gene, *TaPUB1*, enhanced the drought tolerance of transgenic plants, probably by maintaining high water retention and strong antioxidant capacity. The antioxidant capacity might be one of the main mechanisms that enhanced the drought tolerance of the OE plants.

## Materials and Methods

### Plant materials and growth and stress conditions

The transgenic *N*. *benthamiana* plants containing the *35S::TaPUB1* vector (OE9, OE10, OE11) and the WT plants were used in this study. The seeds of *N*. *benthamiana* were sterilized with 70% alcohol for 2 min, treated with 4% sodium hypochlorite solution for 8 min, and then rinsed 5 times with sterilized water and grown, thereafter, in Petri dishes containing MS medium. The *N*. *benthamiana* seeds were sown in roseite in a growth room at 25 °C, under a 16-h light/8-h dark cycle with 200 μmol m^−2^ s^−1^ photon flux density. For the drought and MV treatments, 6-weeks-old seedlings were grown in solutions containing 10% PEG6000 (w/v) or 100 mM MV, respectively. To analyze their drought tolerance, *N*. *benthamiana* plants were grown under normal water conditions for 6 weeks and then were deprived of irrigation completely for 5 or 7 d.

### Measurement of E3 ligase activity

0.1 g fresh leaves were ground with PBS (PH 7.2–7.4). Then according to the Plant E3/UBPL ELISA kit (Shanghai Kenuodi Biological Technology Co. Ltd.) instructions, we add 50 μl of sample to the sample port. Next add 100 μl of enzyme conjugate to standard wells and sample wells except the blank well and incubate for 60 min at 37 °C. The following wash the Microtiter Plate 4 times. Then Add Substrate A 50 μl and Substrate B 50 μl to each well. After incubate for 15 min at 37 °C, add 50 μl Stop Solution. Last, read the Optical Density (O.D.) at 450 nm using a microtiter plate reader within 15 min.

### RNA extraction and cDNA synthesis

To analyze the response of overexpression*-TaPUB1* plants to water stress, we extracted the total RNA from the two-month-old *N*. *benthamiana* leaves after drought treatment or normal water condition using Trizol reagent (TaKaRa, Japan) according to the operation instructions and performed the reverse transcription as described above. 2 μg total RNA was subjected to synthesize cDNA using the HiFiScript Quick gDNA Removal cDNA Kit (Cwbiotech, China) according to the kit instructions. Then, the RT-qPCR was performed with a Bio-Rad CFX96TM real-time PCR system and TransStart Green qPCR SuperMix (TransGen Biotech, China). The PCR thermal cycle is programmed as follows reaction conditions: 94 °C for 60 s, 40 cycles at 94 °C for 15 s, 56 °C for 30 s, then 72 °C for 15 s. The *N*. *benthamiana* β-actin gene was used as reference and the real-time PCR reaction primers are listed in Table 1.

**Table 1:** Primers used in this study.

Sequence of the primers used in PCR analysis	
The cloning of full-length cDNA	
TaPUB1-F	ATGATCTGCGCGATCTCCGG
TaPUB1-R	TTGCGCCGCTGACTCTGATTTTGC
Sequence of the primers used in qRT-PCR analysis	
q-TaPUB1-F	AAATCTCCAGTCATCCACTTCAC
q-TaPUB1-R	CCATCTTCATTACCTTGCCATAC
Nbβ-actin-F	TGGACTCTGGTGATGGTGTC
Nbβ-actin-R	CCTCCAATCCAAACACTGTA
NbCAT-F	GACATCACCTTACCT
NbCAT-R	GAAGTTGTTCCCTACCAGAT
NbGST-F	ACCCTTACCTTTCCCTCA
NbGST-R	TTCCTTCACAGCAGCATC
NbAPX-F	GGATTGGTTGCTGTTGAA
NbAPX-R	CTTGAGGTAGGAGTTGTCG
NbSOD-F	CAACTCCACGGCTTCCAGAC
NbSOD-R	TGGGTCCTGATTAGCAGTGGT
NbLEA-F	CCTTACTCTGTTCCTATTCC
NbLEA-R	TTCTTCCTGATGCTATTACC
NbRbohA-F	ACACACGCCATCAGAACTCCA
NbRbohA-R	CCCACCCAACCAAAATACGC
NbDREB-F	GAATAACCCCAAGAGGCG
NbDREB-R	AGTCAGCGAAGTTCAAGCAA
NbERD-F	CACTGATAAGAACTATGCGTTCAC
NbERD-R	CTAAGCTAATCACATTCAGCGAG
NbNCED-F	CGACCCACGAGTCCAGATTTC
NbNCED-R	GAGCCTAGCAATTCCCGAGTG
NbP5CS-F	AGAGGTGATGGAAGATTAGC
NbP5CS-R	CCAACTGACCGAATAACG

### Measurement of photosynthetic parameters and chlorophyll content

The photosynthetic parameters of adult tobacco lines were measured by a portable photosynthetic system (CIRAS-2 Hitchin, USA). The measurements were carried out under the condition of a CO_2_ concentration of 360 μl l^−1^, PFD of 800 μmol m^−2^ s^−1^, relative humidity of 60–70% and the temperature of the leaf chamber was 25 °C. First, all lines were lighted at least 30 min to induce the stomata open, then lighted at 800 μmol m^−2^ s^−1^ PDF for 15 min to be acclimated.

About 0.1 g fresh leaves of each line were placed in 95% alcohol, and then extraction 40 h in darkness. The quantification was examined with a spectrophotometer. The chlorophyll content was calculated according to the method described of Kim and Kim^47^.

### Measurements of leaf water loss, relative water content, osmotic potential, proline content, and soluble sugar contents

The two-month adult WT and OE lines without water in a greenhouse for 5 days. The leaf water loss rate kinetics was implemented refer to Li *et al*.^48^. The osmotic potential of the leaves was assayed as described by Michel *et al*.^49^. The leaves relative water content were measured as described by González and González-Vilar^50^, the same the proline content and soluble sugar were measured according to the method of Hui *et al*.^51^. RWC computational formula: RWC (%) = (FW − DW)/(TW − DW) × 100. FW: fresh weight, TW: turgid weight, DW: dry weight.

### MDA content and relative electrical conductivity

The MDA levels and relative electrical conductivity were measured based on description of Li *et al*.^48^.

### Western blot analysis

Total protein was extracted from *N*. *benthamiana* leaves using protein extraction buffer^52^. The proteins separated by SDS-PAGE were electrotransfered to a PVDF membrane (Millipore, Billerica, MA) and then, incubated with 2,4-dinitrophenylhydrazine (DNPH, 0.1 mg ml^−1^) dissolved in 2 M HCl. Thereafter, anti-DNP antibody was used to detect the level of protein carbonylation.

### Histochemical ROS staining, measurements of H_2_O_2_ content and O_2_^·−^ production rate

Hydrogen peroxide (H_2_O_2_) and superoxide (${{\rm{O}}}_{2}^{\cdot -}$) accumulation were dected with the staining methods described by Lu *et al*.^53^. Quantitative measurements of H_2_O_2_ and ${{\rm{O}}}_{2}^{\cdot -}$ accumulation followed the methods described by Hui *et al*.^51^ and Wang *et al*.^54^.

### Assay of antioxidant enzyme activity

The determination of SOD, CAT, POD and APX activities were measured as previously described^55^. The Shimadzu (UV-2550) spectrophotometer (Shimadzu, Tokyo, Japan) was used to spectrophotometric analyses.

### Statistical Analysis

Each experiment included three repetition. The experimental data analysis was performed using the data processing system (Zhejiang University, China). Statistical significance was tested using Duncan’s test at the 0.05 and 0.01 probability levels.

## Electronic supplementary material

Supplementary Figure
