# Supplementary material for: Wheat TaPUB1 modulates plant drought stress resistance by improving antioxidant capability
Source: Sci Rep. 2017 Aug 8;7:7549. doi: 10.1038/s41598-017-08181-w (PMC5548723; doi:10.1038/s41598-017-08181-w)
Supplement: Supplementary file 1 — Supplementary Figure [file 41598_2017_8181_MOESM1_ESM.doc]

**Supplementary Figure**

**Wheat *TaPUB1* modulates plant drought stress resistance by improving antioxidant capability**

Guangqiang Zhang^1^, Meng Zhang^1,2^, Zhongxian Zhao^1^, Yuanqing Ren^1^, Qinxue Li^1^ Wei Wang^1^*


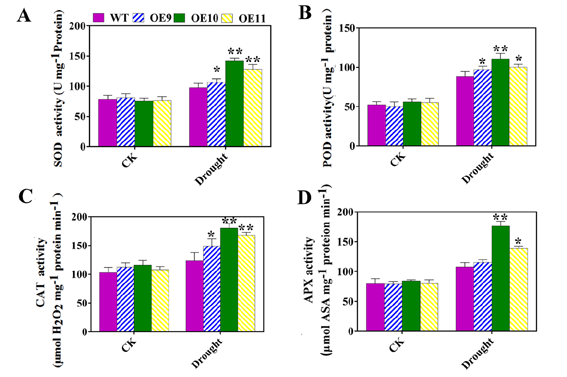


**Supplementary Figure. S1.** **Activities of the antioxidant enzymes in the OE and WT plants under drought treatment**

**(A)** SOD. **(B)** POD. **(C)** CAT. **(D)** APX. The experiments were repeated three times and the bars indicate SEs. * and ** indicate significant differences at P < 0.05 and P < 0.01 among the three overexpression lines and the WT plants.


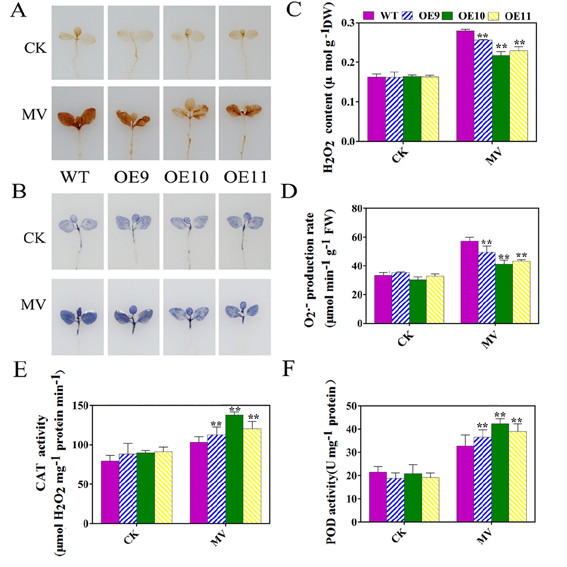


**Supplementary Figure. S2. ROS accumulation and the activity levels of antioxidant enzymes in the transgenic and WT plants under oxidative stress**

**(A)** DAB staining. **(B)** NBT staining. **(C)** Quantification of H_2_O_2_ content. **(D)** Quantification of O_2_^•−^ production rate. **(E)** CAT activity. **(F)** POD activity. The experiments were repeated three times and the bars indicate SEs. * and ** indicate significant differences at P < 0.05 and P < 0.01 in the values among the three overexpression lines and the WT plants.
